# ASO Author Reflections: Identifying Patients Before Treatment Pathways Break Down: From Risk Factors to Actionable Care Pathways

**DOI:** 10.1245/s10434-026-20481-y

**Published:** 2026-08-24

**Authors:** Ioannis Liapis, Manish Tripathi, Nritya Nair, Marcus P. Sirianno, Annabelle L. Fonseca

**Affiliations:** 1 Institute of Cancer Outcomes and Survivorship, University of Alabama at Birmingham, Birmingham, AL USA; 2 Department of Surgery, University of Alabama at Birmingham, Birmingham, AL USA; 3 Collat School of Business, University of Alabama at Birmingham, Birmingham, AL USA

## Past

Receipt of guideline-concordant treatment (GCT) is associated with improved survival in patients with pancreatic ductal adenocarcinoma (PDAC), yet over one-third of patients do not receive it.^1^ Prior studies have identified demographic, clinical, and socioeconomic factors associated with non-receipt of GCT using conventional regression approaches.^2,3^ Although these studies have identified important risk factors, they provide limited insight into how these factors interact in clinical practice or whether different patients follow distinct pathways leading to non-receipt of GCT. Understanding these pathways may identify clinically recognizable patient groups at increased risk for non-receipt of GCT and help guide interventions before treatment opportunities are lost.

## Present

Among 1040 patients with  PDAC treated at two academic centers, one-third did not receive GCT.^4^ Non-receipt of GCT most commonly resulted from clinical deterioration during treatment, health system and access barriers, and loss to follow-up. Patients who failed to receive GCT did not represent a single high-risk population but instead followed distinct clinical pathways. Classification tree analysis demonstrated that the pathways leading to non-receipt of GCT differed according to patients’ socioeconomic context, with neighborhood deprivation consistently representing the earliest point of stratification. Among patients residing in higher-deprivation neighborhoods, non-receipt of GCT was more commonly associated with factors related to entry into the healthcare system, including emergency department-initiated workup. In contrast, among patients residing in lower-deprivation neighborhoods, treatment delivery was more frequently associated with age, functional status, and social support. These findings suggest that non-receipt of GCT occurs through distinct pathways that may require different intervention strategies. Importantly, the analysis was intentionally restricted to variables available at the initial oncology consultation. As a result, the identified pathways represent factors that are recognizable before the start of treatment, creating an opportunity for earlier intervention rather than retrospective identification of patients who ultimately do not receive GCT.

## Future

Our findings suggest that early identification of patients at increased risk for non-receipt of GCT may provide an opportunity to implement targeted supportive interventions before treatment opportunities are lost. Improving receipt of GCT will likely require interventions that are matched to the pathways through which patients fail to complete treatment rather than a single uniform approach. Future studies should prospectively validate these findings and evaluate whether early identification of patients at increased risk for non-receipt of GCT can facilitate targeted interventions that improve delivery of stage-appropriate pancreatic cancer care. Ultimately, the value of clinically interpretable prediction models will depend not on their ability to classify patients, but on whether they enable interventions that improve delivery of GCT and patient outcomes.^5^
